# Entropy Sharing in Ransomware: Bypassing Entropy-Based Detection of Cryptographic Operations

**DOI:** 10.3390/s24051446

**Published:** 2024-02-23

**Authors:** Jiseok Bang, Jeong Nyeo Kim, Seungkwang Lee

**Affiliations:** 1 Department of Cyber Security, Dankook University, Yongin 16890, Republic of Korea; jsbang.kevin@gmail.com; 2 Cyber Security Research Division, Electronics and Telecommunications Research Institute (ETRI), Daejeon 34129, Republic of Korea

**Keywords:** ransomware, cryptographic operation, entropy, detection, neutralization

## Abstract

This study presents a groundbreaking approach to the ever-evolving challenge of ransomware detection. A lot of detection methods predominantly rely on pinpointing high-entropy blocks, which is a hallmark of the encryption techniques commonly employed in ransomware. These blocks, typically difficult to recover, serve as key indicators of malicious activity. So far, many neutralization techniques have been introduced so that ransomware utilizing standard encryption can effectively bypass these entropy-based detection systems. However, these have limited capabilities or require relatively high computational costs. To address these problems, we introduce a new concept *entropy sharing*. This method can be seamlessly integrated with every type of cryptographic algorithm and is also composed of lightweight operations, masking the high-entropy blocks undetectable. In addition, the proposed method cannot be easily nullified, contrary to simple encoding methods, without knowing *the order of shares*. Our findings demonstrate that *entropy sharing* can effectively bypass entropy-based detection systems. Ransomware utilizing such attack methods can cause significant damage, as they are difficult to detect through conventional detection methods.

## 1. Introduction

### 1.1. Ransomware Attacks: Economic Impact and Phases

Ransomware is a significant cybersecurity concern, specifically crypto-ransomware, which infects computers, encrypts files, and demands a ransom for decryption. It has emerged as a prominent and financially devastating form of cybercrime, with the estimated global cost projected to reach approximately $265 billion annually by 2031 (https://cybersecurityventures.com/global-ransomware-damage-costs-predicted-to-reach-250-billion-usd-by-2031 (accessed on 4 November 2023)). This escalation in financial losses underscores the urgent need to understand the economic impact of ransomware attacks. High-profile incidents like WannaCry and Petya have contributed to the growing global damage caused by ransomware attacks.

In general, ransomware attacks typically unfold through a series of distinct phases. Four fundamental steps capture the essential characteristics of ransomware attacks: Initial compromise, Establishing foothold, Encryption, and Extortion. (1) The initial compromise phase marks the point at which a ransomware attack infiltrates the first computer. Attackers employ various methods to deliver and execute the initial compromise, including phishing, spear phishing, corrupted web pages, and the exploitation of security vulnerabilities and system misconfigurations. (2) Following the initial compromise, attackers typically aim to establish a permanent foothold within the compromised system and move laterally within the network. This phase often involves connecting to command and control (C&C) servers, which are internet hosts or entire infrastructures designed to control the behavior of ransomware. These servers issue commands, generate and distribute encryption keys, collect information about victims, and store critical data related to the ransomware attack. However, some ransomware attacks do not rely on C&C infrastructure and instead limit themselves to host detection capabilities. (3) The encryption phase consists of several stages, including key generation, searching for target files with specific extensions, encryption, and potential deletion or overwriting of backups. Ransomware employs different encryption methods, such as symmetric and asymmetric ciphers. Symmetric encryption is favored for its speed in encrypting large data volumes, while asymmetric encryption protects the symmetric key. Ransomware employs tactics like overwriting or renaming original files, saving encrypted files in new locations, or temporarily moving and restoring files during encryption. (4) Once the files are fully or partially encrypted, ransomware enters the extortion phase. During this phase, ransomware creates a ransom note, typically in the form of a text or HTML file, providing instructions to the victim on how to retrieve their data.

### 1.2. General Approaches to Ransomware Detection

Detecting ransomware during its encryption phase can be achieved through various methods, each focusing on different aspects of cryptographic operations. One prevalent method involves monitoring API and system calls. This technique examines the use of encryption-related API calls, encompassing processes like encryption, file manipulation, and key management. The incorporation of machine learning has significantly refined this approach, allowing for more nuanced detection of encryption activities through pattern recognition in API and system call usage.

Another key strategy is I/O monitoring. This method analyzes I/O requests related to memory, file systems, and network operations, aiming to detect ransomware encryption by identifying anomalous patterns and behaviors. It typically utilizes a combination of classifiers and analyzes various features from the network and data flow to pinpoint potential threats.

Monitoring the file system is also a crucial technique in detecting ransomware. This approach involves examining changes in the file system’s state and file attributes to spot encryption indicators. Some researchers utilize entropy analysis, searching for files with abnormally high entropy as possible encryption evidence. Alternatively, observing file system events for unusual file operation patterns can also be indicative of ransomware activity.

Entropy is a measure of how unpredictable something is. In cryptography, entropy serves as an indicator of how unpredictable the ciphertext is relative to the plaintext. In this context, the randomness of ciphertext reaches its maximum when the ratio of 0 s to 1 s is equal. In other words, encryption is a procedure that alters information, rendering it more random or introducing uncertainty, thereby inherently increasing entropy. For this reason, blocks written by benign applications in the file system, which do not perform encryption operations, have significantly lower entropy compared to standard encryption with an overwhelming probability [1]. Consequently, the detection of such high-entropy blocks has been a common indicator in ransomware detection methods [2]. From the attacker’s perspective, however, it is possible to bypass these detection systems using neutralization techniques that lower the entropy of encrypted files. Common methods for this include base64 encoding, format-preserving encryption (FPE), and intermittent encryption. While these methods can make encrypted files more recoverable, they also present limitations in terms of efficiency. For a more detailed discussion on these neutralization techniques, refer to Section 2.3.

### 1.3. Contributions

The next generation of ransomware could potentially evade current detection methods by using encryption techniques that produce moderate-level entropy. However, selecting an algorithm that reduces the ciphertext’s entropy might not align with ransomware business objectives, as it could increase the chances of successful decryption of the plaintext. In this paper, we introduce an efficient and effective method for bypassing ransomware detection. Our approach presents a new threat model for ransomware, which leverages standard encryption algorithms. This model is designed to maintain the balance between evading detection and preserving the robustness of the encryption, thereby adhering to the core goals of ransomware operations. The main contributions of this paper are as follows:

- We propose an entropy reduction technique, aptly named ***entropy sharing***, that can be applied to the output of both symmetric and asymmetric encryption algorithms commonly utilized in ransomware. Before introducing *entropy sharing*, we also outline a basic concept of simple bit decomposition aimed at achieving minimal entropy levels.
- Through the frequency test defined in the NIST randomness test suit, we demonstrate that the proposed method can effectively bypass existing entropy-based detection techniques.
- We present a decoding approach named ***entropy recomposition***, which is designed to be applied to the output of *entropy sharing*. This process is followed by decryption, facilitating the restoration of the victim’s files. Unlike other encoding methods, a distinctive feature is that decoding is impossible if the order of entropy shares is unknown.
- We evaluate the overhead of the proposed method when combined with encryption algorithms and assess their impact on the total computation time. The results show that there is minimal change in the efficiency of ransomware attacks, allowing for the rapid corruption of a large number of files.

The rest of this paper is structured as follows: Section 2 offers a comprehensive overview of current ransomware detection and neutralization strategies. Section 3 details our innovative method designed to obscure cryptographic operations in ransomware. This involves a novel encoding technique that transforms high-entropy blocks into blocks with lower or medium entropy. In Section 4, we present our experimental findings. These experiments demonstrate the effectiveness of *entropy sharing* in ransomware encryption and evaluate the additional overhead incurred. Our primary approach for assessing entropy randomness involves the use of NIST frequency tests, which are specifically applied to the data written on the file system. Section 5 focuses on analyzing the results of *entropy recomposition* at different ratios, which are aimed at countering ransomware that employs *entropy sharing*. This section also explores the entropy characteristics of write blocks following simple bit decomposition and evaluates the accuracy in distinguishing between encrypted and non-encrypted files using Shannon entropy values. The paper concludes with Section 6, summarizing our findings and contributions.

## 2. Ransomware Detection and Neutralization Methods

Numerous studies have focused on addressing the growing threat of ransomware, typically dividing their approaches into two primary categories: prevention and detection. Prevention strategies aim to either reduce the impact or stop an attack in its early stages. Among these, regular backups are frequently noted as the most effective way to minimize ransomware damage. However, even backups can fall prey to encryption, rendering file recovery extremely challenging without a decryption key. Due to the ineffectiveness of cryptanalysis against sophisticated encryption techniques, the emphasis increasingly shifts to detection strategies categorized into two types: process-centric methods and data-centric methods.

### 2.1. Process-Centric Methods

Process-centric detection involves monitoring specific activities or behaviors in executing programs, such as encryption key generation or the use of cryptography-related APIs, which are commonly associated with ransomware. These activities form the basis for building event-based detection systems. Alternatively, machine learning-based classification models can be developed by observing malicious process behavioral patterns in run-time data.

#### 2.1.1. Event-Based Detection

Event-based detection revolves around tracking specific indicators of an impending ransomware attack. For instance, Ahmed et al. [3] recommended monitoring traffic behaviors or domain-generating algorithms (DGAs) that provide new domains as needed. Andronio et al. [4] proposed the Heldroid method for tracing threatening messages retrieval from the C&C server, which is typically not included in the ransomware payload. Palisse et al. [5] suggested tracking Microsoft’s cryptographic APIs, commonly used in many ransomware types, to prevent victims’ file encryption.

However, this detection method has limitations, including the requirement for prior knowledge about encryption technologies used by different ransomware families. Advanced ransomware can function independently without internet connectivity or C&C server assistance, meaning encryption keys or data retrieval may not occur during the attack. Furthermore, these methods may have a high false alarm rate since benign programs may also employ the observed events, leading to increased false alerts. As previously mentioned [6], API hooking as a ransomware detection method can be undermined by copying a DLL containing the desired code and dynamically loading it into the process with a different name. Additionally, ransomware can bypass API hooking by using customized cryptosystems instead of standard APIs to encrypt user files.

#### 2.1.2. Machine Learning Implementation for Detection

Machine learning techniques have become increasingly popular in ransomware detection research, offering effective means to identify malicious patterns [7,8,9,10,11,12,13,14,15,16,17,18,19]. These studies have employed a range of classification algorithms to pinpoint ransomware attack signatures. Classifiers can be divided into two types: singular and ensemble. Singular classifiers use a single machine learning algorithm for classification, whereas ensemble classifiers integrate several algorithms to collaboratively perform the task [20,21,22,23]. Examples of singular classifiers are support vector machines, logistic regression, decision trees, and deep neural networks. In contrast, ensemble classifiers include techniques like bagging, adaboost, and random forests. Ensemble learning combines the outputs of multiple singular or base classifiers to arrive at a final decision. The application of machine learning in ransomware detection is typically segmented into two categories: delayed detection and early detection.

Delayed detection involves analyzing comprehensive runtime data generated during the execution of a malicious program to train detection models. Various methods, such as Bayesian networks and statistical approaches, have been used to detect ransomware based on CPU, I/O, memory usage, network traffic, or data from physical sensors within computers. However, delayed detection relies on complete data and may fail to detect an attack before data encryption begins. On the other hand, early detection aims to identify ransomware threats before the data encryption process starts. Techniques such as using a fraction of the initial data or a fixed duration threshold during ransomware execution have been proposed. However, early detection based on limited data can result in lower accuracy rates.

### 2.2. Data-Centric Methods

Data-centric ransomware detection involves monitoring the targets of ransomware rather than the malicious activities initiating the attack [24]. Extensive research has been conducted on data-centric methods for identifying ransomware [25,26,27,28,29,30]. The primary objective is to identify abnormal modifications through continuous analysis of user documents, with metrics such as entropy and similarity typically used for this purpose.

One straightforward approach involves using decoy files, also known as honey files, to detect malevolent alterations of user documents, as demonstrated by Moore [31]. These decoy files, integrated within the user’s system, enable the identification of changes to user data, as legitimate programs do not need to access them. Similarly, Song et al. [32] suggested analyzing key user data locations using decoy files. Gomez-Hernandez and Alvarez-Gonzalez [33] implemented decoy files in the target environment, aiming to stop the ransomware process upon its interaction with these files. Mehnaz and Mudgerikar [34] also used the decoy approach for early ransomware detection and prevention. Moreover, relying solely on decoy-based detection does not ensure that ransomware will target the decoy files first, thereby placing the victim’s data at considerable risk [35,36].

Entropy has been widely used as a metric in data-centric approaches since it tends to rise when a file is encrypted. Numerous studies have utilized entropy calculations, such as Shannon entropy, which quantifies data uncertainty, to identify ransomware threats [35]. For example, Nolen Scaife’s team [37] used Shannon entropy to examine modifications in files when accessed. The Shannon entropy of a byte array can be computed using the formula:

$$e=\sum_{i=0}^{255}P_{i}\log_{2}\frac{1}{P_{i}}$$

Here, $P_{i}$ is the relative frequency of a byte value *i* occurring in the array, given by $F_{i}/n$, where *n* is the total bytes to be analyzed, and $F_{i}$ is the number of appearance of *i* such that $n=\sum_{i=0}^{255}F_{i}$. The computed result ranges from 0 to 8, with 8 denoting a perfectly balanced distribution of byte values in the array. Due to the uniform probability distribution in encrypted files, they often approach the maximum entropy value of 8. The method uses statistical analysis to identify changes in a user’s file structure before and after access and also employs a similarity metric based on the concept that successful encryption results in a distinctly different version of the file.

Kharraz et al. proposed a comparable detection method, called UNVEIL [6], where they examined the dynamic I/O buffer content and measured the difference in Shannon entropy between read and write operations. In addition to analyzing the generic I/O access patterns of ransomware, they identified two indicators of ransomware detection: a significant increase in entropy between read and write data buffers at a specific file offset or the creation of new high-entropy files. This observation is crucial because, even when ransomware overwrites original files with low entropy blocks to securely delete them, it must generate an encrypted version of the original files. This process inevitably leads to the generation of high-entropy data during ransomware attacks.

Similarly, REDEMPTION [38], like UNVEIL, calculates the Shannon entropy of the data buffers associated with each read and write request to a file. By comparing the entropy values of read and write requests from the same file offset, it becomes a powerful indicator of ransomware activity. REDEMPTION calculates a malicious score for each process that requests privileged operations, including factors such as the ratio of modified blocks in a file and an increase in entropy, as a true positive signal of ransomware detection. Therefore, an increase in entropy can be considered an important metric for ransomware detection. However, here we note that just relying on the calculated Shannon entropy value to distinguish between encrypted and non-encrypted files would be a difficult task generating a lot of false positives and negatives [39]. In Section 5, we show our experimental result on this issue.

The NIST randomness test suite can also be used for a similar purpose to identify suspicious cryptographic operations that result in the writing of high-entropy blocks in the file system, as in the case of Rcryptect [1]. This test suite includes various tests developed to assess the randomness of binary sequences. The entropy of binary sequences is tested based on the assumptions of uniformity and scalability. The test suite compares the test statistic value computed on the target binary sequence to a critical value determined from a reference distribution of the statistic under the assumption of randomness. If the test statistic value surpasses the critical value, the null hypothesis (H0) that the sequence is random is rejected. Otherwise, H0 is accepted. For instance, the frequency test provides the most basic evidence of non-randomness and is used to assess entropy levels in this case. Algorithm 1 explains the frequency test taking a byte sequence *buf* with *size* bytes; Table 1 summarizes the notation used in the algorithm. Contrary to Shannon entropy, the frequency test can distinguish between non-encrypted and encrypted blocks with an overwhelming probability [1].
**Algorithm 1:** Frequency test defined in NIST randomness test suit
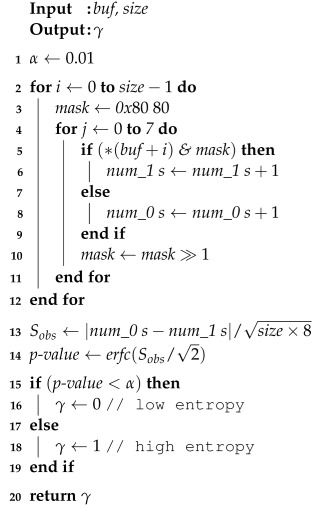


In summary, data-centric methods for ransomware detection focus on monitoring the targets of ransomware, using metrics like entropy to identify abnormal modification. However, ransomware continues to evolve in ways that can bypass these detection methods. This will be examined in more depth in the following section.

### 2.3. Neutralization Methods

Known ransomware variants apply various methods to conceal the high entropy of encrypted files to circumvent entropy-based detection techniques. The most notable methods include encoding techniques such as Base64 and ASCII85 encoding. These encoding methods can reduce the entropy of a ciphertext to a level similar to plaintext. Most of these encodings consist of lightweight operations, thereby not significantly impacting the speed of the ransomware attack. However, if the defense system identifies that the file is encoded, it can effectively detect the file infected with ransomware because decoding can be performed without the key [40].

As an alternative, FPE can be utilized. FPE is an encryption method that maintains the same format for plaintext and ciphertext, thus keeping the entropy after encryption similar to that of plaintext. In [40], the FF1 algorithm was used to circumvent entropy-based ransomware detection using the characteristics of FPE. However, this can reduce the speed of ransomware’s encryption attack due to its high computational complexity. More specifically, either FF1 or FPEs based on prefix cipher, cycle-walking cipher, and generalized-Feistel cipher involve repeated execution of block ciphers like AES in their internal operations to preserve the format of the plaintext. Therefore, compared to the encoding-based neutralization methods, it significantly reduces the efficiency of ransomware attacks.

Another neutralization method is the use of intermittent encryption. This method encrypts only parts of a file, reducing the increase in entropy of the file after encryption. However, to enhance the efficiency of ransomware attacks using intermittent encryption, the more the encryption area is reduced, the greater the possibility of file restoration becomes. In other words, leaving a large portion of the files unencrypted means that for some file formats, we can extract data from the non-encrypted parts of the files and recover some of the data from there [41]. On the other hand, as the proportion of the encrypted area within a file increases, the overall entropy also rises, thereby heightening the likelihood of detection.

## 3. Hiding Cryptographic Operations

To address the limitations of previous neutralization techniques, we introduce a novel ransomware model incorporated by our encoding method that converts blocks with high entropy, generated by standard cryptographic operations, into larger blocks with lower entropy. By doing so, ransomware can effectively bypass existing entropy-based detection techniques. To achieve this, the proposed encoding technique transforms encrypted blocks into blocks with an average entropy level according to the type of the original file.

### 3.1. Simple Bit Decomposition for Lowest Entropy

Before going into depth, we show a simple encoding method, providing the lowest entropy level. For a given standard block cipher E, let P be the plaintext and C be the ciphertext. Then, we have C = E(P), where P and C are *n* bytes in length. Let C = $C_{1}∥C_{2}∥\ldots ∥C_{n-1}∥C_{n}$, where the symbol ∥ means byte concatenation.

Due to the essential property of standard block ciphers, C presents high entropy with overwhelming probability. For each subbyte $C_{i}$, we can encode it as the lowest entropy blocks of *n* × 8 bytes in length. If we denote

$$C_{i}\mapsto b_{0}\;b_{1}\;b_{2}\;b_{3}\;b_{4}\;b_{5}\;b_{6}\;b_{7},$$

this gives us:

$$\begin{array}{c} \begin{array}{cccccccccc} C_{i}\left[0\right] & \mapsto & \;b_{0} & \;0 & \;\;0 & \;\;0 & \;\;0 & \;\;0 & \;\;0 & \;0\\ C_{i}\left[1\right] & \mapsto & \;0 & \;b_{1} & \;\;0 & \;\;0 & \;\;0 & \;\;0 & \;\;0 & \;0\\ \; & \; & & \; & & \;\;\vdots & \; & & \\ C_{i}\left[7\right] & \mapsto & \;0 & \;0 & \;\;0 & \;\;0 & \;\;0 & \;\;0 & \;\;0 & \;b_{7} \end{array} \end{array}$$

This can be simply generalized as follows:

$$C_{i}\left[j\right]=\left\{b_{j}\ll \left(7-j\right)\right\},\mathrm{where}\;j\in \left[0,7\right].$$

Here, the crucial observation is that, as shown in Section 4, this simple encoding can be easily defeated by detecting frequent appearances of lowest-entropy blocks in the file systems. In the following, our proposed encoding method solves this problem by converting high-entropy blocks of cryptographic outcomes into mid-level blocks of benign files.

### 3.2. Proposed Scheme

The ransomware under consideration in our study exhibits several key characteristics: Firstly, it employs standard cryptographic functions for encrypting files. Secondly, the entropy level of the blocks written to files during an attack is neither excessively high nor low, closely mirroring that of a benign file. Thirdly, the process employed to reduce entropy does not substantially affect the speed of the encryption attack. Last but not least, the decoding process must be dependent on secret information. To satisfy these characteristics, we propose an advanced encoding technique, named ***entropy sharing***. This method will be elaborated on in the following.

#### 3.2.1. Entropy Sharing

**Figure 1:**
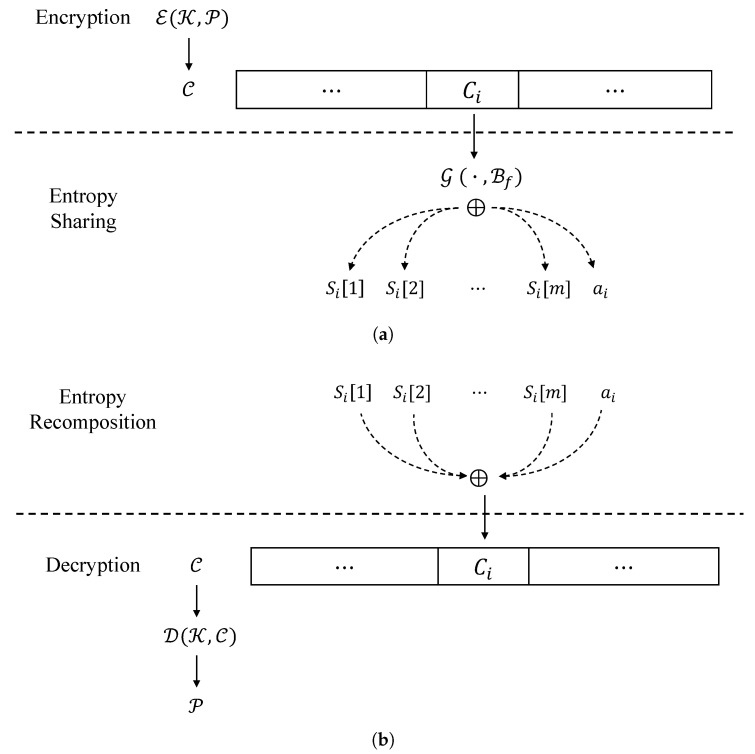
Overview of ransomware attack (**a**) and restore (**b**) using entropy sharing and recomposition, respectively. (**a**) Entropy sharing following encryption; (**b**) entropy recomposition followed by decryption.

Figure 1 illustrates the overall ransomware attack and restoration procedures, incorporating the proposed encoding and decoding techniques. Table 2 provides a summary of descriptions for each notation, which will be used throughout this paper. In particular, Figure 1a demonstrates how *entropy sharing* converts a high-entropy ciphertext into a byte sequence with benign-level entropy. For a secret key denoted by K and a plaintext denoted by P, respectively, E represents a standard block cipher such as the AES algorithm used by ransomware to encrypt victim’s files. While ransomware typically writes the resulting C to the file system, the new ransomware threat model utilizes *entropy sharing*, a post-processing step that transforms high-entropy blocks into benign-level ones, to bypass detection methods based on entropy.

**Figure 2:**
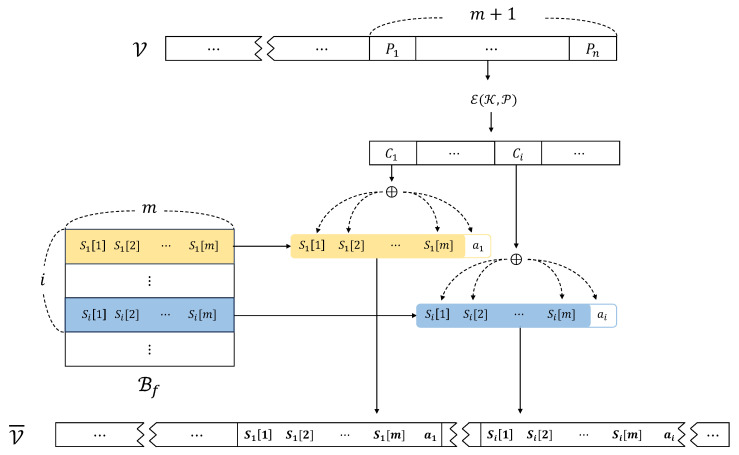
G’s operation generating benign-level entropy using $C_{i}$ and $B_{f}$ as inputs.

For an *n*-byte ciphertext C computed by E(K,P), *entropy sharing* takes each subbyte $C_{i\in \{1,n\}}$ and divides it into *m* + 1 shares. Then, we have an n×m byte stream consisting of *n* sequences of $S_{i}\left[1\right]||S_{i}\left[2\right]||\ldots ||S_{i}\left[m\right]$, which exhibits a non-random distribution, thereby providing a benign-level of entropy. To achieve this purpose, let us assume that there exists a generator $G(C_{i},B_{f})$, which splits $C_{i}$ into *m* + 1 shares ($S_{i}\left[1\right],S_{i}\left[2\right],\ldots ,S_{i}\left[m\right],a_{i}$) such that

$$C_{i}=\overset{m}{\underset{j=1}{⨁}}S_{i}\left[j\right]⊕a_{i},$$

where $B_{f}$ is a reference file packaged within the ransomware. In other words, G extracts a byte stream with a benign level of entropy from $B_{f}$ and splits $C_{i}$ into the *m* + 1 shares. Finally, *entropy sharing* replaces $C_{i}$ with the G’s output and writes it to the victim’s file. Figure 2 describes this overall process of *entropy sharing*.

Ransomware today often encrypts only a specific set of file types that are commonly used and vital in both personal and business settings. Attackers use encryption to take crucial data hostage, demanding ransom from victims for decryption keys. By focusing on these particular file formats, ransomware aims to impact many users, thereby increasing the probability of receiving ransom payments. Let F represent this set of file types, including {.jpg, .pdf, .pptx, .docx, .mp3, .mp4, .txt, .zip, etc.}. When a file of type f∈F is targeted, G uses a reference file $B_{f}$ to produce an entropy level similar to what is typically seen in files of type *f*.

In simpler terms, G sequentially reads *m* bytes from $B_{f}$ for each $C_{i}$. Considering that the size of ransomware-targeted files might be larger than that of the benign files $B_{f}$, these are handled as if they were in a circular queue-like structure. Since $a_{i}$ is not predominant in terms of entropy within the entire *m* + 1 bytes, the resulting encoded output exhibits the entropy levels of benign files. This similarity poses a significant challenge for current detection methods to distinguish between files held hostage by ransomware and original files (detailed discussion in Section 4).

#### 3.2.2. Entropy Recomposition

Suppose that the victim pays the ransom for restoring the encrypted files. In this case, a restoration process may be expected to recover the encrypted files (in reality, only 54% of victims reportedly paid the ransom and got data back. “The state of ransomware 2023”, A SOPHOS whitepaper, May 2023). This can be achieved through the proposed ***entropy recomposition***. Figure 1b demonstrates how *entropy recomposition* restores C from G’s output followed by decryption P = D(K,C).

**Figure 3:**
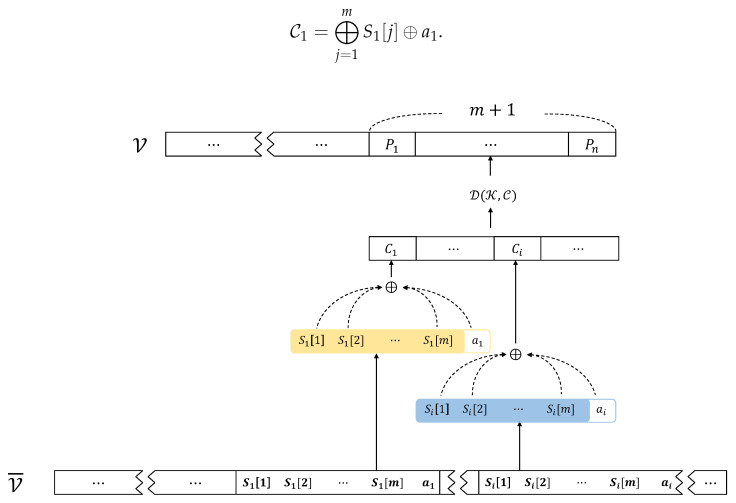
Restoring a victim’s file using entropy recomposition and decryption.

For a corrupted file $\bar{V}$ which is attacked by *entropy sharing*, this can be grouped into *m* + 1 byte units. Then, restoring V from $\bar{V}$, shown in Figure 3, can be performed as follows. First, *m* + 1 bytes, say ($S_{1}\left[1\right]$, $S_{1}\left[2\right]$, …, $S_{1}\left[m\right]$, $a_{1}$), are read from the victim’s encrypted file $\bar{V}$. Second, obtaining $C_{1}$ can be performed by *recomposition* as follows:

$$C_{1}=\overset{m}{\underset{j=1}{⨁}}S_{1}\left[j\right]⊕a_{1}.$$

Next, the *n*-byte ciphertext C can be obtained by repeating *n* times. Lastly, D(K,C) gives us P.

The proposed scheme involves simple XOR operations to the existing standard cryptographic functions and thus has little impact on the computational cost of ransomware operation. In the following section, we will provide a more detailed explanation based on various experiments.

## 4. Evaluation

In this section, we investigate the impact of *entropy sharing* on encrypted samples by using the AES-128 algorithm. We omit experiments involving other cryptographic algorithms for *entropy sharing* and *recomposition*, as various standard ciphers, including asymmetric key algorithms, used in ransomware, are known to produce similar entropy patterns in their blocks [1]. Our analysis focuses on assessing the pass rate and *p*-values of the frequency test for the original files, the resulting ciphertexts, and their encoded outputs obtained through *entropy sharing*. Furthermore, we provide an evaluation of the computational costs involved. Please take note of the analysis of the impact of simple bit decomposition in Section 5.2. In a concise summary, it is observed that simple bit decomposition yields negligible *p*-values due to the encoding of each byte in 8-byte values, which possess the HW of only 8.

### 4.1. Experimental Environment and Methods

Based on a Windows 11 host machine featuring an AMD 8-Core Processor with a clock speed of 3.4 GHz and 32 GB of RAM, the following experiments were conducted on an Ubuntu 22.04 guest operating system. This was achieved through the utilization of VMware Workstation 17 Player, which assigned 4 cores and 16 GB of RAM.

Consider the set F = {*mp3, jpg, pdf, zip*}, for which we collected 100 sample files for each type f∈F with each sample ranging in size from 1 to 20 MB. The frequency test conducted in our experiments follows the outlined procedure. Each sample file was read in binary mode and divided into 100 binary sequences. For each binary sequence, the frequency test was carried out with a significance level of α = 0.01. Under NIST SP 800-22 [42], if 96 or more out of the 100 binary sequences are determined to be random, the sample file can be classified as random. To visualize the results of the frequency test, we calculated the pass rate and the average *p*-values on the 100 binary sequences of each file. To provide a complete view of the results for the pass rates and average *p*-values of individual files across the 100 samples for each type, we present a graphical representation using box plots, displaying the five-number summary: the minimum, the maximum, the sample median, and the first and third quartiles.

Based on this, $B_{f}$ files were prepared for each type *f* to demonstrate the outcomes of the proposed *entropy sharing*. The detailed experimental procedure performed on 100 samples for each type is as follows:

1. Conduct a frequency test on an original (non-encrypted) sample file.
2. Encrypt the original file using the AES-128 algorithm in ECB mode. Perform a frequency test on the resulting ciphertext.
3. Apply *entropy sharing* to encode the mentioned ciphertext. For each type, $B_{f}$ is employed, and the order of entropy shares is defined as *m* ∈ {0, 1, 3, 5, 7, 9}. Notably, when *m* = 0, it signifies the encrypted file without *entropy sharing*. Once the outcomes for each order are acquired, proceed to conduct the frequency test.
4. To assess the impact of the secret keys input into AES, the same experiment is replicated using eight distinct secret keys, as shown in Table 3.

### 4.2. Experimental Results on Entropy Sharing

Moving forward, we present a range of experimental findings concerning *entropy sharing* and the frequency test. As previously explained, four samples were prepared as $B_{f}$, where f∈F, and they show the pass rate and the average *p*-values on the frequency test as shown in Table 4. In light of these outcomes, these benign files can be utilized to contribute a benign level of entropy, serving as non-random samples for each type *f*.

**Figure 4:**
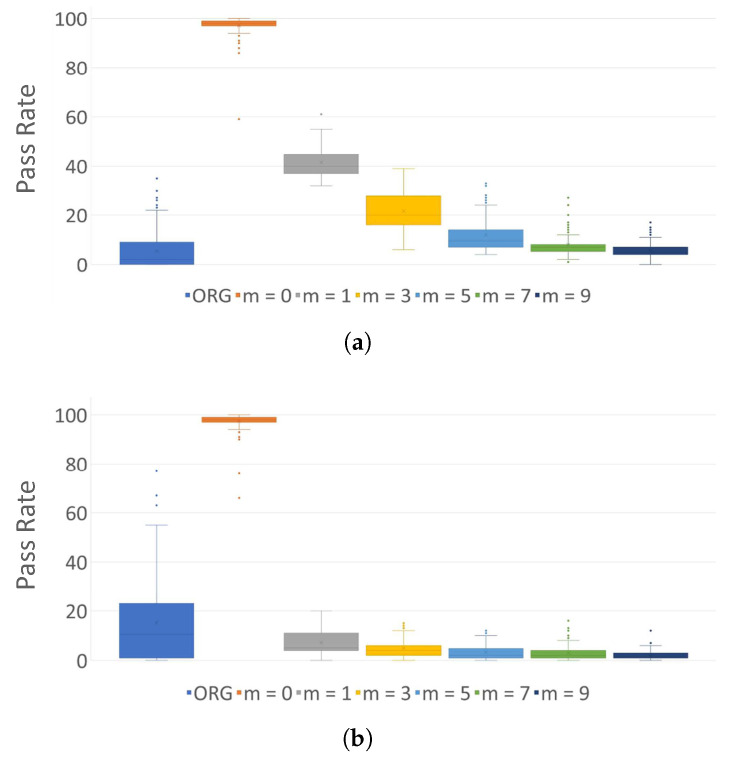

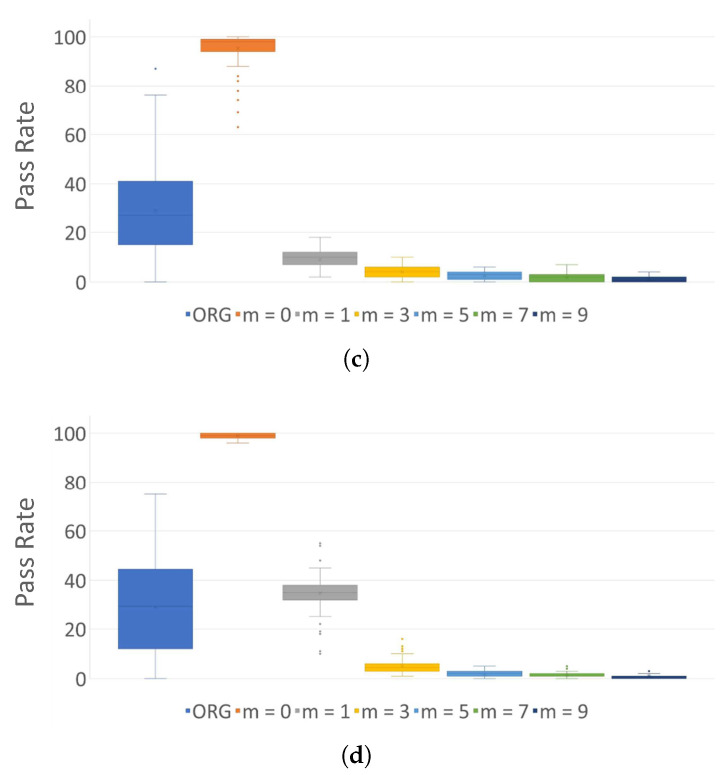
Pass rates of the frequency test on 100 binary sequences across four sample types using K#1. ORG: original sample files. Here, *m* = 0 represents encrypted files. (**a**) mp3; (**b**) jpg; (**c**) pdf; (**d**) zip.

**Figure 5:**
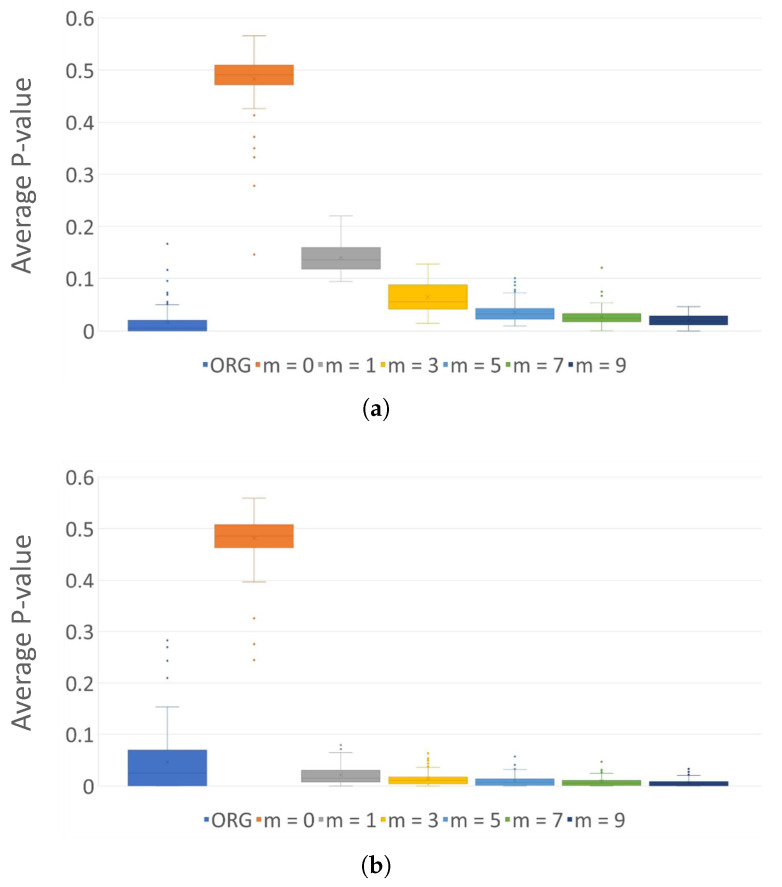

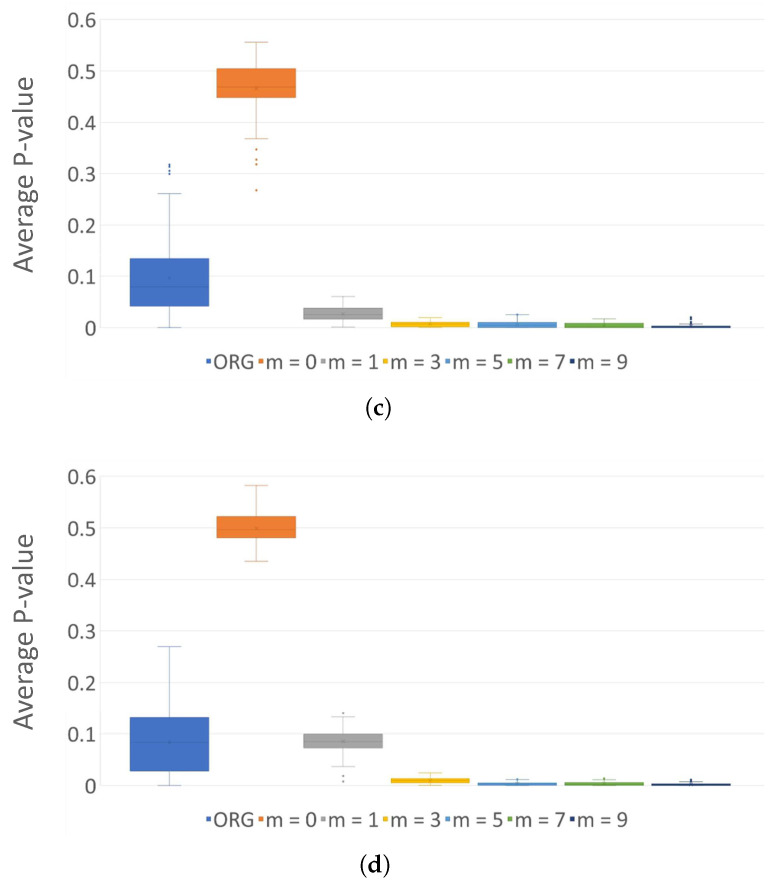
Average *P-values* for the frequency test on 100 binary sequences across four sample types using K#1. (**a**) mp3; (**b**) jpg; (**c**) pdf; (**d**) zip.

**Figure 6:**
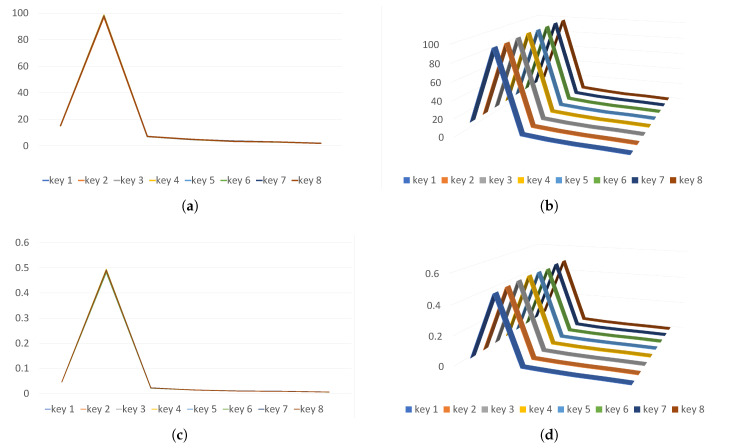
Average pass rates and *p*-values for frequency test on 100 binary sequences for each JPG sample file using each of 8 different keys. (**a**) Overlapping average pass rates; (**b**) average pass rates; (**c**) overlapping average *p*-values; (**d**) average *p*-values.

The original files within set F are determined to exhibit non-random results in the frequency test. However, when subjected to encryption using the AES-128 algorithm, they transform into random binary sequences regardless of the file type. Nevertheless, *entropy sharing* on the encrypted samples reveals a significant reduction in entropy. Figure 4 and Figure 5 depicts the pass rates and average *p*-values of the frequency test for each type of original sample files, encrypted files under K#1, and the encoded outputs across different orders, respectively. Note that a comprehensive collection of pass rates, obtained by applying eight distinct secret keys, can be located in Appendix A. Notably, as the order *m* of entropy shares increases, the entropy diminishes visibly. This observation underscores that *entropy sharing* can effectively circumvent existing entropy-based detection of cryptographic operations in ransomware. Figure 6 presents the average pass rates on the outcomes of applying *entropy sharing* to encrypted files when different secret keys, as shown in Table 3, are injected into AES. Figure 6a illustrates this in a three-dimensional representation, while Figure 6b projects the results onto a two-dimensional plane by overlaying the eight graphs. An intriguing observation here is that despite changing the secret key, there is a slight variation in the pass rates.

### 4.3. Computational Costs

We validate the impact of *entropy sharing* on encryption speed, thereby examining its effect on the speed of ransomware attacks. To achieve this, the following experiments were conducted:

1. Measure the latency when operating the AES-128 algorithm in ECB mode for each file. In this case, *m* = 0.
2. Measure the time taken for encryption and encoding to complete for $m_{i}$ ∈ {1, 3, 5, 7, 9}.
3. Divide the size of the encrypted or the encrypted and encoded result file into 16-byte blocks to measure the increase in attack time for a single block.
4. Calculate the ratio for each order as the elapsed time for $m_{i}$ divided by the elpased time for *m* = 0.

Upon decrypting either the encrypted file or the file corrupted using both encryption and *entropy sharing*, we also experimentally verify the time required for restoration via decryption and subsequent *entropy recomposition*. The results will contribute to a comprehensive understanding of the practical implications of using *entropy recomposition* for data recovery.

As indicated in Table 5, *entropy sharing* results in a mere 1% overhead, while *entropy recomposition* introduces an additional time of less than 1%, in comparison to encryption and decryption, respectively. Notably, the computational expenses of *entropy recomposition* exhibit a tendency to diminish as the order of shares *m* increases. This can be attributed to the reduction in the number of XOR operation loops during the decoding process, wherein, in each loop iteration, *m* + 1 bytes are consolidated into a single byte, which is a process that becomes more noticeable as the value of *m* increases.

## 5. Discussion

In this section, we explore the effectiveness and limitations of *entropy recomposition* as a countermeasure against *entropy sharing* in ransomware. We note the pitfalls of simple bit decomposition in reducing file entropy. Additionally, we discuss the difficulty in distinguishing encrypted from non-encrypted files using Shannon entropy. Finally, we consider the memory requirements and detection issues related to ransomware attacks that utilize *entropy sharing*.

### 5.1. Entropy Recomposition at Arbitrary Ratios for Counteracting Entropy Sharing

Up to this point, we have demonstrated the effectiveness of *entropy sharing* as a means to effectively evade entropy-based ransomware detection techniques. In light of the emergence of such novel threats, let us delve into the discussion of entropy-based detection methods aimed at preemptively countering these challenges.

**Figure 7:**
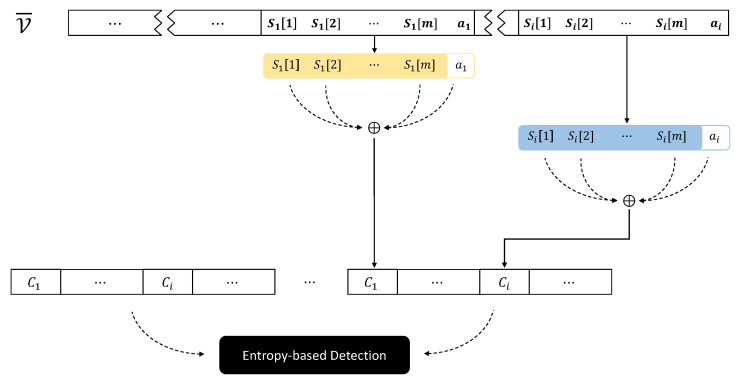
Illustration of potential detection scenario for ransomware cryptographic operations with entropy sharing.

As seen in the decoding process facilitated by *entropy recomposition*, if the order of entropy shares *m* can be accurately inferred, it would be feasible to detect the cryptographic operations of ransomware protected by *entropy sharing*. This detection could be achieved through compression by XORing *m* + 1 bytes into a single byte for every writing block in the file system, as illustrated in Figure 7.

However, there are two key considerations to address in this approach. First, due to the unknown order of entropy shares selected by ransomware, accurately deducing it proves challenging, requiring the use of an arbitrary compression ratio *r*:1 for *recomposition*. While a ratio of *r* = *m* + 1 has a high likelihood of detecting cryptographic operations by decoding input blocks back to their original ciphertext, different scenarios where *r* ≠ *m* + 1 require empirical investigation to understand their implications. Second, although encoded blocks from *entropy sharing* tend to exhibit higher entropy when compressed correctly, the impact of entropy on non-encrypted files also becomes crucial under arbitrary compression ratios of *r*:1. If this leads to the generation of blocks with increased entropy, it can lead to a rise in false positives, subsequently thereby affecting the overall accuracy of the detection system.

To address these concerns, a series of experiments were conducted. For each original file among the set of 100 sample jpg files, we performed the following procedures for various *r* ∈ {2, 4, 6, 8, 10}:

- XOR compression was applied with a *r*:1 ratio.
- AES encryption using K#1 was performed, followed by *r*:1 XOR compression.
- Entropy sharing was applied with *m* = 3 after the AES encryption using K#1, followed by *r*:1 XOR compression.

The average of the pass rates for the frequency test was computed for each case.

**Figure 8:**
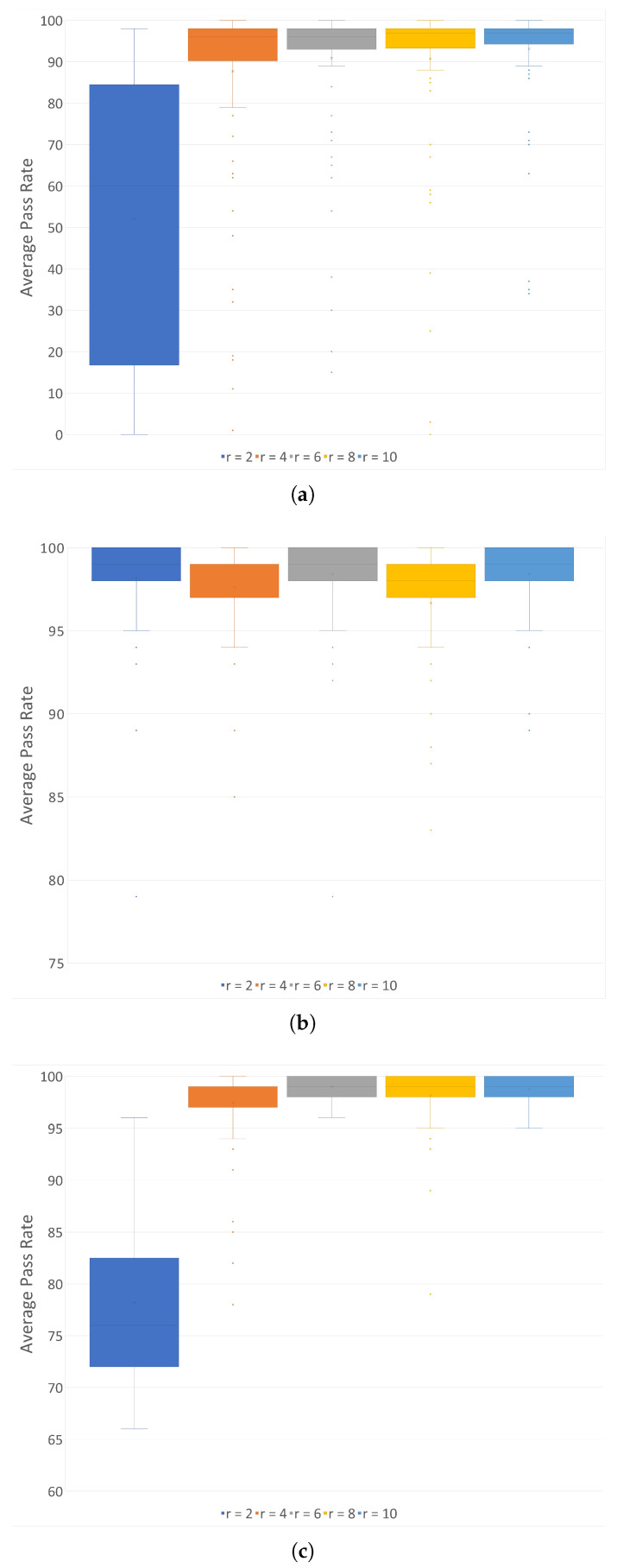
Average pass rates of frequency test vs. compression ratios *r* on original, encrypted, and encoded files. (**a**) ORG; (**b**) *m* = 0; (**c**) *m* = 3.

The experimental results, depicted in Figure 8, yield several key insights: Encrypted files (where *m* = 0) display randomness independent of the compression ratio *r*. For encoded outcomes (where *m* = 3), randomness is observed when *r* ≥ *m* + 1. Non-encrypted original files (ORG) start exhibiting a significantly higher level of randomness beginning at a compression ratio *r* = 4. Notably, even at *r* = 2, some blocks are already identified as random. This suggests that during *recomposition* at a given ratio *r*, if *r* = *m* + 1, the encoded blocks are accurately decoded back to blocks of the original ciphertext. However, as *r* increases, the non-encrypted blocks exhibit increasingly higher entropy, leading to a significant false positive rate. For this reason, the inability to ascertain the exact order of entropy sharing renders precise decoding unfeasible and results in a high rate of false positives, thereby hindering the effective operation of ransomware detection systems.

### 5.2. Simple Bit Decomposition: Implications and Experiments

In Section 3.1, we proposed simple bit decomposition as the most straightforward approach to significantly decrease the entropy of encrypted files. However, as mentioned earlier, this encoding method excessively diminishes the entropy of the output, potentially rendering it susceptible to detection through entropy-based cryptographic operation analysis aimed at identifying low-entropy blocks. We aim to present experimental results to illustrate this effect.

To this end, we conducted frequency tests on a total of 400 samples across the four previously mentioned types, following AES encryption and the subsequent application of simple bit decomposition. The experimental results revealed that for all files, the pass rate was consistently 0, and *p*-values, being so small that it could not even be represented as a floating-point number, consistently resulted in a value of 0. This signifies that the application of simple bit decomposition to the encrypted results leads to an effectively negligible level of entropy.

### 5.3. False Positives and Negatives Related to Shannon Entropy

As detailed in Section 2, the attempt to distinguish encrypted and non-encrypted files through Shannon entropy calculation for a designated block encounters challenges of false positives and false negatives. To directly validate this assertion, we subjected the previously utilized samples to encryption and *entropy sharing*, subsequently computing the Shannon entropy.

The obtained experimental results are presented in Table 6. Taking into account the standard deviation for each scenario, it becomes evident that calculating the Shannon entropy value does not yield a distinct demarcation between non-encrypted original files and encrypted (and encoded) files.

### 5.4. Issue on Additional Memory Requirement

There are two main types of ransomware attack methods. The first involves overwriting the original with the encrypted result, while the second involves creating new files and storing the encrypted data there. In the latter case, the original files are either deleted or overwritten with meaningless values, making recovery impossible.

One consideration when ransomware conducts an attack through *entropy sharing* is the increase in memory space occupied by the attacked files. The most significant increase occurs when encrypting the entire file; if the attack is conducted with the given order of shares *m*, the size of the ciphertext can increase by up to *m* + 1 times. To reduce such an increase in file size, intermittent (or partial) encryption can be applied. When overwriting the ciphertext with increased size onto the original, there is a risk of losing yet-to-be-encrypted original blocks. To address this issue, it is possible to read the plaintext blocks in advance before encryption or append the latter part of ciphertext blocks exceeding the size of plaintext blocks to the end of the file.

To prepare for the scenario where ransomware is detected through the pattern of increasing the original file’s size, it is also possible to write the latter parts of ciphertext blocks exceeding the size of plaintext blocks in a separate file. In this case, additional metadata need to be provided during the *entropy recomposition* process to indicate how each ciphertext block should be combined with a specific original file. Since this I/O pattern is not easy to be distinguishable from legitimate file operations (due to the benign level of entropy), checking I/O access patterns and entropy leads to a decrease in the true positive rate for detecting ransomware.

## 6. Conclusions and Future Work

Existing ransomware may perform standard encryption operations, not lightweight encryption, to reduce the possibility of decryption. As a result, high entropy inevitably occurs when ransomware operates. Accordingly, entropy-based detection techniques have intensively used the high entropy occurring during the operation of ransomware. The existing methods of neutralization techniques for bypassing entropy-based detection have limited efficiency and destructive effects of ransomware. In this study, we demonstrated that even ransomware applying standard encryption can effectively neutralize the numerous entropy-based detection techniques proposed so far. Specifically, we proposed an encoding technique called *entropy sharing*, which strongly reduces the encryption operation result to the benign level of entropy that a non-encrypted regular file has, and proved that the computational cost it imposes is very low and does not significantly affect the cost of ransomware attacks. We also proposed a decoding method called *entropy recomposition* as an inverse operation of *entropy sharing*. Here, *entropy recomposition* requires knowledge of the order of entropy sharing to enable successful decoding. Through a series of experiments employing the frequency test as defined in NIST SP 800-22, we have demonstrated that *entropy sharing* can effectively circumvent current entropy-based ransomware detection methods. This is achieved by presenting non-randomness in cryptographic operations across various sample files. These findings highlight the necessity for a new, reliable run-time detection system capable of countering potential ransomware threats. Our future research will focus on developing innovative detection techniques that are robust against both existing ransomware variants and the novel threat model introduced in this paper. 

## Figures and Tables

**Table 1:** Notations used in Algorithm 1.

Notation	Description
α	Significance level set to 0.01, indicating a 1% probability threshold for the test
buf	Input buffer containing the binary sequence to test
size	The number of elements in *buf*
mask	Binary mask initialized to *0x*80 to isolate bits in a byte
num_0 s	Counter for the number of 0 s in the sequence
num_1 s	Counter for the number of 1 s in the sequence
$S_{obs}$	Computed statistic for the observed discrepancy between the number of 0 s and 1 s
p−value	The probability that the observed balance of 0 s and 1 s could occur by chance
γ	Frequency test result; 0 for imbalance, 1 for balance.

**Table 2:** Notations used in *entropy sharing*.

Notation	Description
P	Plaintext
C	Ciphertext
K	Secret key
E(K,P)	Encryption taking K and P
D(K,C)	Decryption taking K and C
$B_{f}$	Benign file, where f∈F
G	Byte-sequence generator of benign-level entropy taking a subbyte of C and $B_{f}$
*m*	Order of entropy shares
V	Victim’s original file
$\bar{V}$	Victim’s encrypted file

**Table 3:** Secret keys used in the AES-128 algorithm.

	K
1	0x67C6697351FF4AEC29CDBAABF2FBE346
2	0x7CC254F81BE8E78D765A2E63339FC99A
3	0x66320DB73158A35A255D051758E95ED4
4	0xABB2CDC69BB454110E827441213DDC87
5	0x70E93EA141E1FC673E017E97EADC6B96
6	0x8F385C2AECB03BFB32AF3C54EC18DB5C
7	0x021AFE43FBFAAA3AFB29D1E6053C7C94
8	0x75D8BE6189F95CBBA8990F95B1EBF1B3

**Table 4:** The frequency test results on $B_{f}$.

*f*	Pass Rate	Average *p*-Values
mp3	5	0.005085
jpg	2	0.006348
pdf	1	0.001249
zip	10	0.026651

**Table 5:** The elapsed time for encryption and decryption for a single block with entropy sharing and entropy recomposition, respectively.

*m*	Entropy Sharing		Entropy Recomposition	
	Elapsed Time (μs)	Ratio	Elapsed Time (μs)	Ratio
0	0.37007	1	0.36518	1
1	0.37396	1.0105	0.36698	1.0049
3	0.37410	1.0108	0.36612	1.0025
5	0.37416	1.0110	0.36563	1.0016
7	0.37431	1.0114	0.36563	1.0012
9	0.37460	1.0122	0.36554	1.0009

**Table 6:** Average and standard deviation (S.D.) of Shannon entropy values for original, encrypted, and encoded files.

	ORG	*m*					
		0	1	3	5	7	9
Shannon entropy avg.	7.935655	7.998874	7.995119	7.98972	7.987368	7.985997	7.985153
S.D.	0.127836	0.005177	0.000277	0.00038	0.000335	0.000238	0.000202

**Figure A1:**
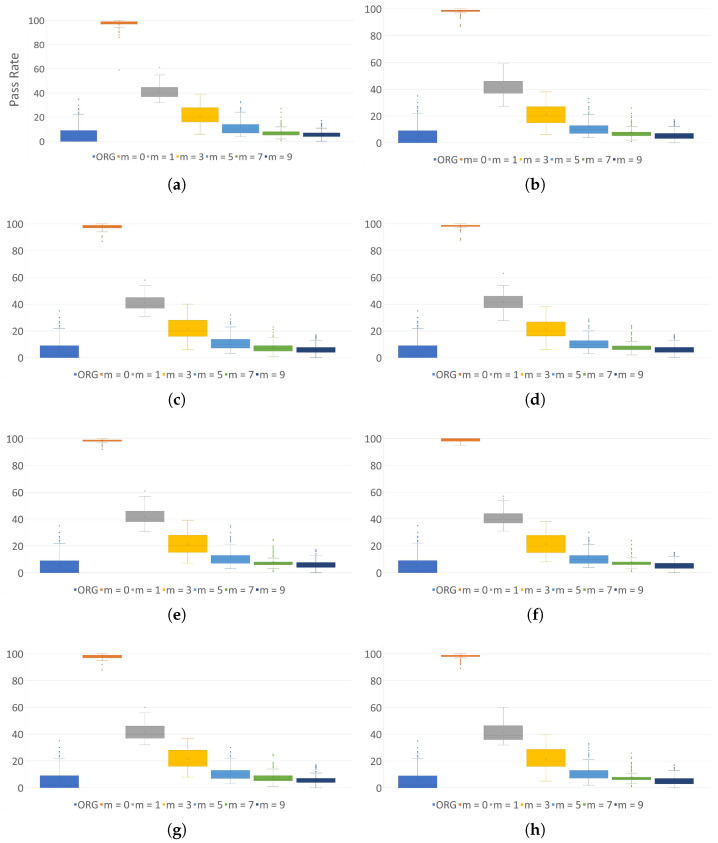
Pass rates of frequency test with 8 different keys on MP3 samples. (**a**) K#1; (**b**) K#2; (**c**) K#3; (**d**) K#4; (**e**) K#5; (**f**) K#6; (**g**) K#7; (**h**) K#8.

**Figure A2:**
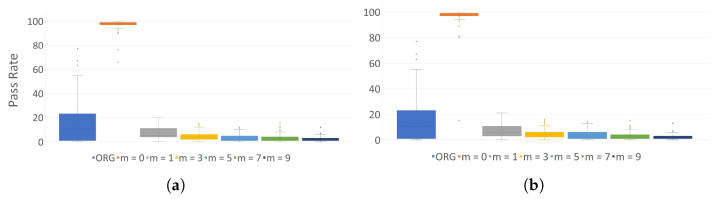

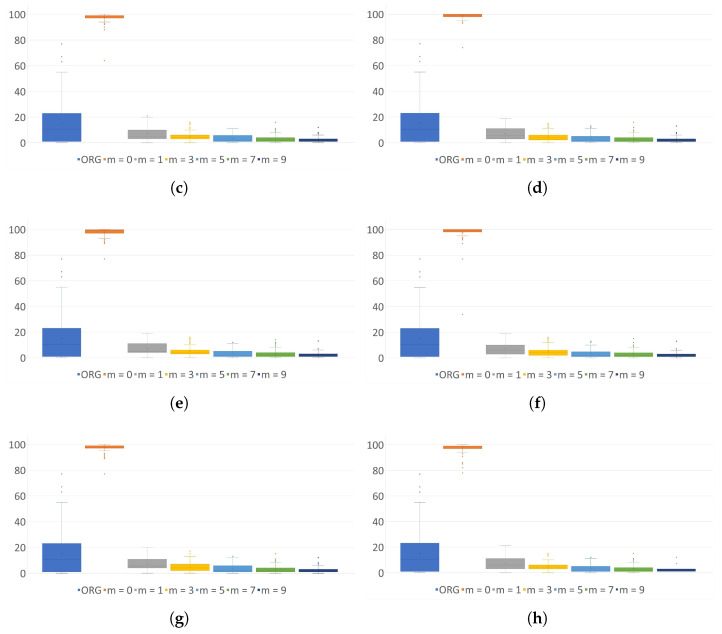
Pass rates of frequency test with 8 different keys on JPG samples. (**a**) K#1; (**b**) K#2; (**c**) K#3; (**d**) K#4; (**e**) K#5; (**f**) K#6; (**g**) K#7; (**h**) K#8.

**Figure A3:**
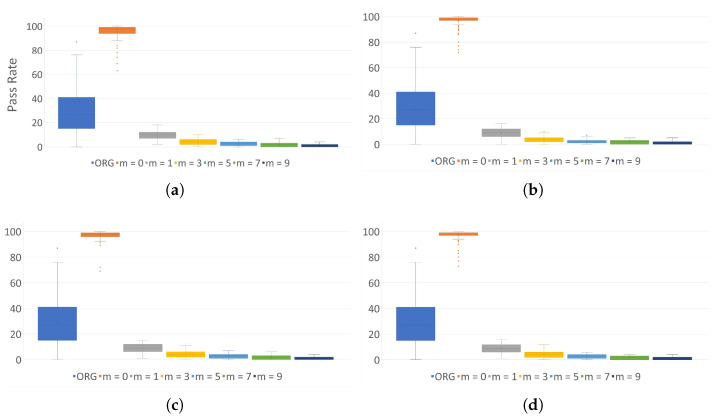

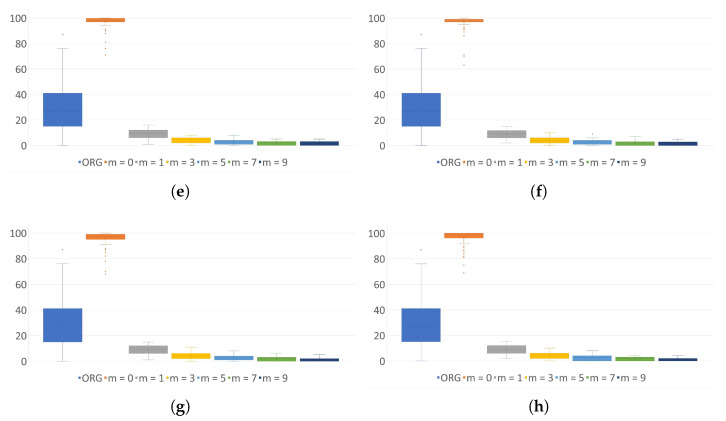
Pass rates of frequency test with 8 different keys on PDF samples. (**a**) K#1; (**b**) K#2; (**c**) K#3; (**d**) K#4; (**e**) K#5; (**f**) K#6; (**g**) K#7; (**h**) K#8.

**Figure A4:**
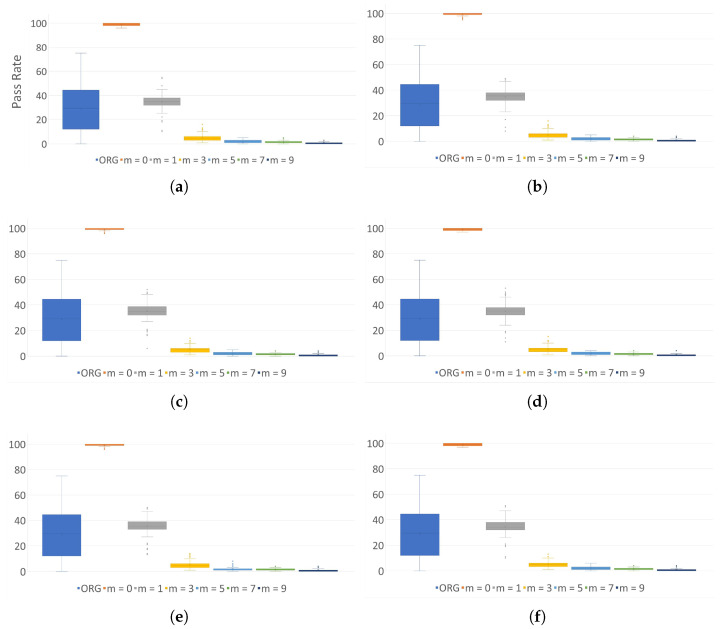

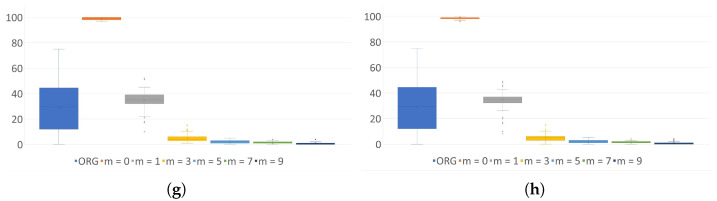
Pass rates of frequency test with 8 different keys on ZIP samples. (**a**) K#1; (**b**) K#2; (**c**) K#3; (**d**) K#4; (**e**) K#5; (**f**) K#6; (**g**) K#7; (**h**) K#8.

**Figure A5:**
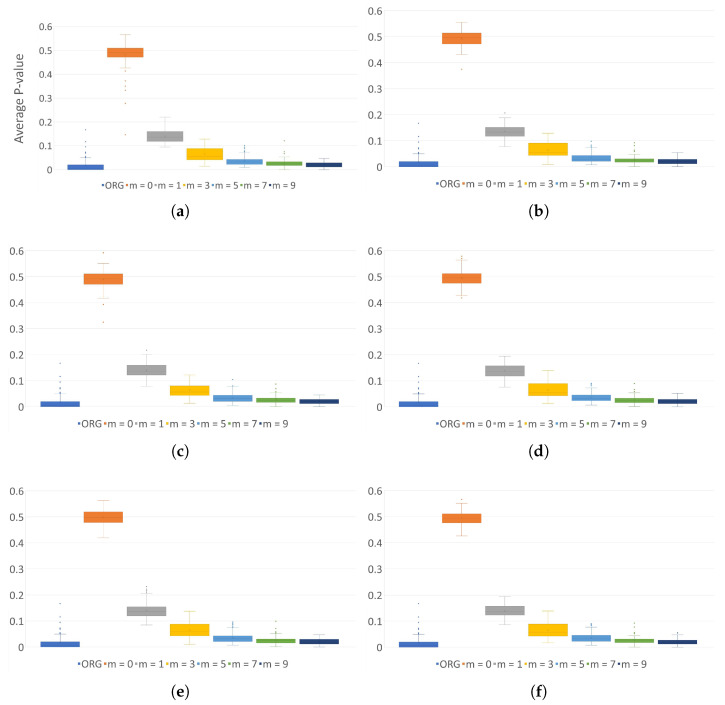

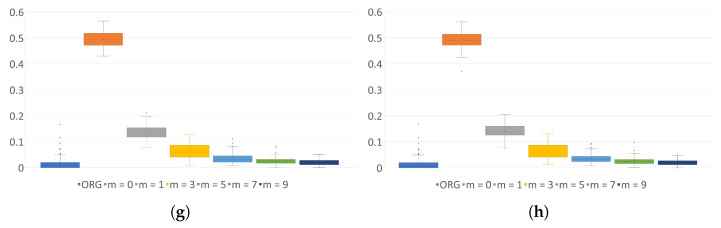
*p*-values averages of frequency test with 8 different keys on MP3 samples. (**a**) K#1; (**b**) K#2; (**c**) K#3; (**d**) K#4; (**e**) K#5; (**f**) K#6; (**g**) K#7; (**h**) K#8.

**Figure A6:**
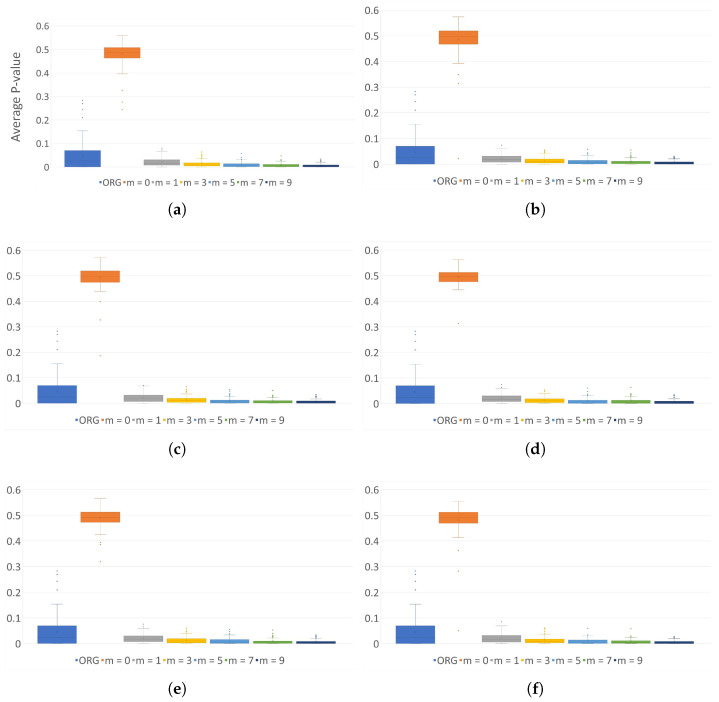

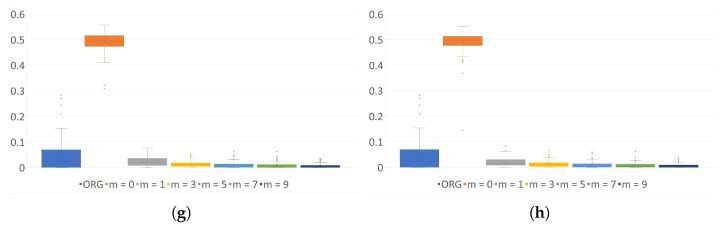
*p*-values averages of frequency test with 8 different keys on JPG samples. (**a**) K#1; (**b**) K#2; (**c**) K#3; (**d**) K#4; (**e**) K#5; (**f**) K#6; (**g**) K#7; (**h**) K#8.

**Figure A7:**
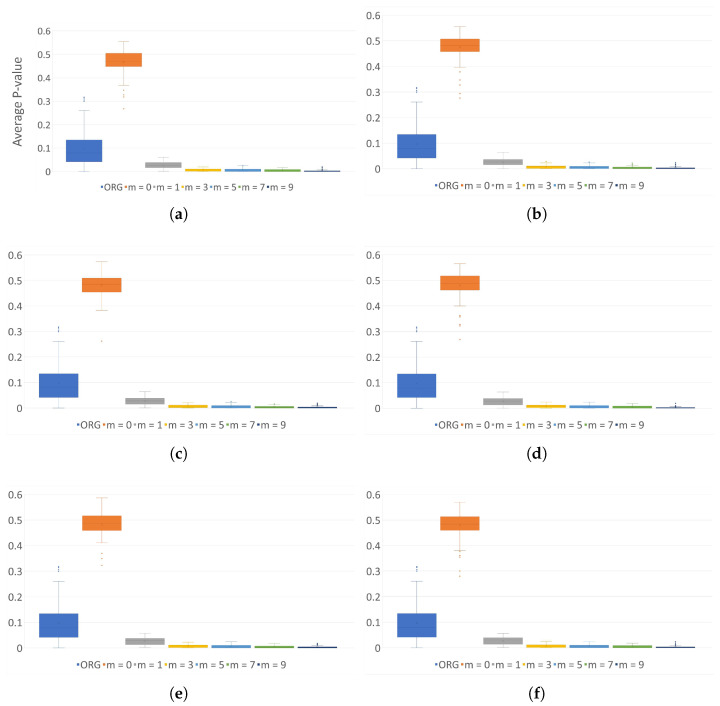

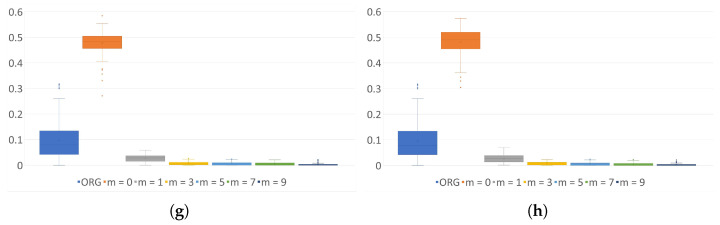
*p*-values averages of frequency test with 8 different keys on PDF samples. (**a**) K#1; (**b**) K#2; (**c**) K#3; (**d**) K#4; (**e**) K#5; (**f**) K#6; (**g**) K#7; (**h**) K#8.

**Figure A8:**
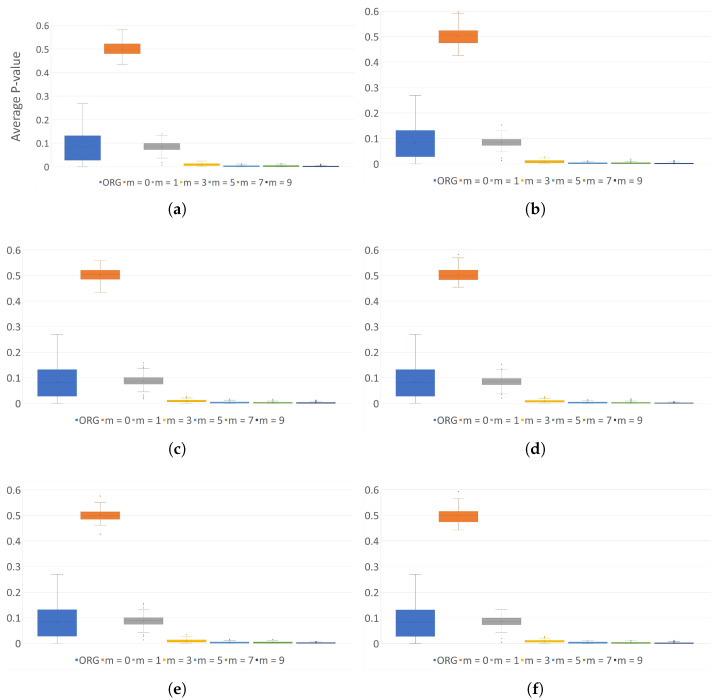

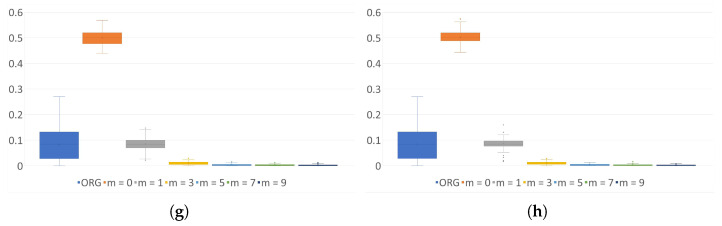
*p*-values averages of frequency test with 8 different keys on ZIP samples. (**a**) K#1; (**b**) K#2; (**c**) K#3; (**d**) K#4; (**e**) K#5; (**f**) K#6; (**g**) K#7; (**h**) K#8.

**Figure A9:**
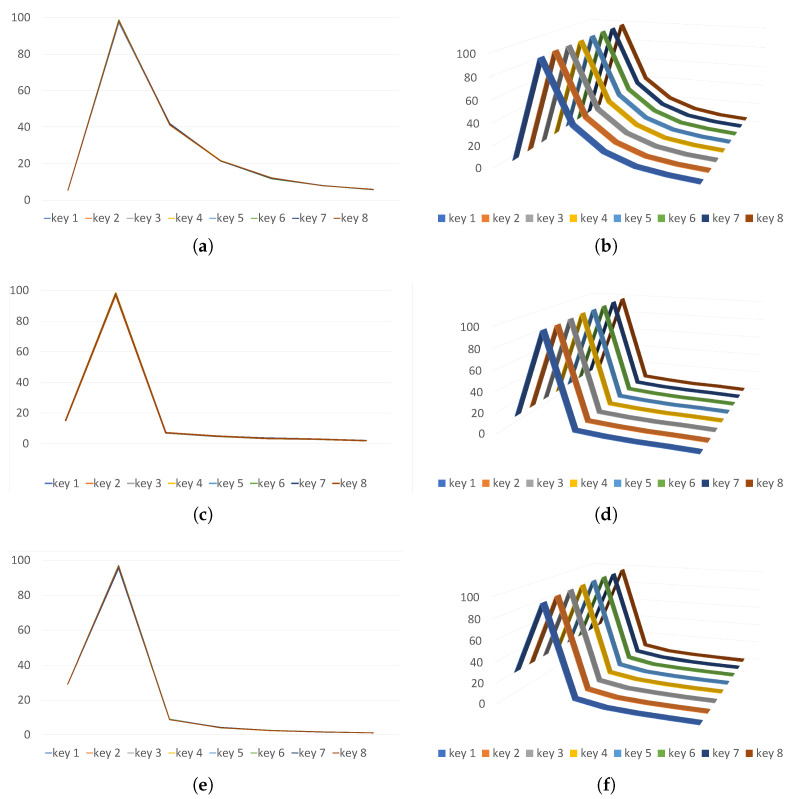

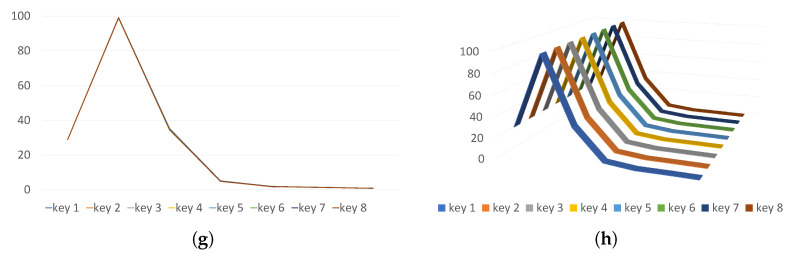
Two-dimensional (2D) and 3D visualization of pass rate averages across 8 different keys for frequency test on each sample type. (**a**) Overlapping average MP3 pass rates; (**b**) average MP3 pass rates; (**c**) overlapping average JPG pass rates; (**d**) average JPG pass rates; (**e**) overlapping average PDF pass rates; (**f**) average PDF pass rates; (**g**) overlapping average ZIP pass rates; (**h**) average ZIP pass rates.

**Figure A10:**
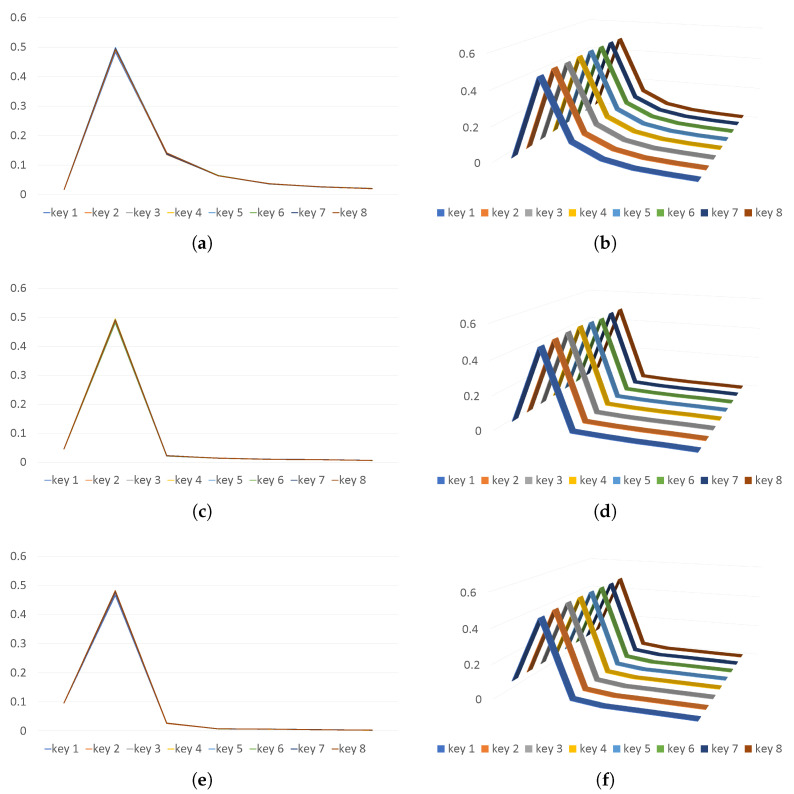

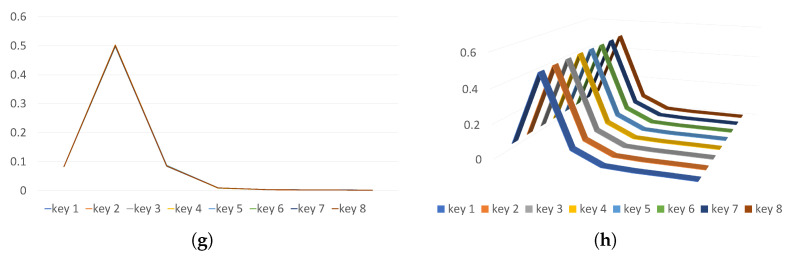
Two-dimensional (2D) and 3D visualization of *p*-values averages across 8 different keys for frequency test on each sample type. (**a**) Overlapping average MP3 *p*-values; (**b**) average MP3 *p*-values; (**c**) overlapping average JPG *p*-values; (**d**) average JPG *p*-values; (**e**) overlapping average PDF *p*-values; (**f**) average PDF *p*-values; (**g**) overlapping average ZIP *p*-values; (**h**) average ZIP *p*-values.

## Data Availability

Data are contained within the article.

## Appendix A. Full Experimental Results for 8 Different Keys

To empirically demonstrate the effects of *entropy sharing*, a series of experiments were conducted involving a collection of 100 files for each of the following types: mp3, jpg, pdf, and zip. For each file type, the original file, encrypted file, and result files for different orders of entropy shares (*m* = 1, 3, 5, 7, 9) were subjected to frequency tests, yielding pass rates and *p*-values. To analyze the influence of encryption keys on encryption, the process was repeated by encrypting with eight distinct random secret keys before analyzing the results of *entropy sharing*. Due to space constraints within the paper, the corresponding box plots illustrating the aforementioned experiments will be included in this appendix.

Figure A1, Figure A2, Figure A3 and Figure A4 show the pass rates, and Figure A5, Figure A6, Figure A7 and Figure A8 illustrate the *p*-values averages of the frequency test for K#1–K#8. As shown in these figures, the experiment results are consistent with the analysis provided in Section 4. Lastly, Figure A9 and Figure A10 tell us that the key has nearly no effect on the pass rates and *p*-values of the frequency test.
